# A method for age-matched OCT angiography deviation mapping in the assessment of disease- related changes to the radial peripapillary capillaries

**DOI:** 10.1371/journal.pone.0197062

**Published:** 2018-05-24

**Authors:** Alexander Pinhas, Rachel Linderman, Shelley Mo, Brian D. Krawitz, Lawrence S. Geyman, Joseph Carroll, Richard B. Rosen, Toco Y. Chui

**Affiliations:** 1 Department of Ophthalmology, New York Eye and Ear Infirmary of Mount Sinai, New York, NY, United States of America; 2 Department of Ophthalmology, State University of New York Downstate Medical Center, Brooklyn, NY, United States of America; 3 Department of Cell Biology, Neurology and Anatomy, The Medical College of Wisconsin, Milwaukee, WI, United States of America; 4 Icahn School of Medicine at Mount Sinai, New York, NY, United States of America; 5 Department of Ophthalmology & Visual Sciences, The Medical College of Wisconsin, Milwaukee, WI, United States of America; Massachusetts Eye & Ear Infirmary, Harvard Medical School, UNITED STATES

## Abstract

**Purpose:**

To present a method for age-matched deviation mapping in the assessment of disease-related changes to the radial peripapillary capillaries (RPCs).

**Methods:**

We reviewed 4.5x4.5mm *en face* peripapillary OCT-A scans of 133 healthy control eyes (133 subjects, mean 41.5 yrs, range 11–82 yrs) and 4 eyes with distinct retinal pathologies, obtained using spectral-domain optical coherence tomography angiography. Statistical analysis was performed to evaluate the impact of age on RPC perfusion densities. RPC density group mean and standard deviation maps were generated for each decade of life. Deviation maps were created for the diseased eyes based on these maps. Large peripapillary vessel (LPV; noncapillary vessel) perfusion density was also studied for impact of age.

**Results:**

Average healthy RPC density was 42.5±1.47%. ANOVA and pairwise Tukey-Kramer tests showed that RPC density in the ≥60yr group was significantly lower compared to RPC density in all younger decades of life (p<0.01). Average healthy LPV density was 21.5±3.07%. Linear regression models indicated that LPV density decreased with age, however ANOVA and pairwise Tukey-Kramer tests did not reach statistical significance. Deviation mapping enabled us to quantitatively and visually elucidate the significance of RPC density changes in disease.

**Conclusions:**

It is important to consider changes that occur with aging when analyzing RPC and LPV density changes in disease. RPC density, coupled with age-matched deviation mapping techniques, represents a potentially clinically useful method in detecting changes to peripapillary perfusion in disease.

## Introduction

First described by Michaelson in 1954, the radial peripapillary capillary (RPC) layer is the most superficial retinal capillary layer that supplies the retinal nerve fibers surrounding the optic nerve head [1, 2]. Henkind and contemporary researchers in the 1960s postulated that these capillaries, having long parallel paths and rare anastomoses, may be more vulnerable to increased intra-ocular pressure (IOP) or hemodynamic disorders, and thus may be affected selectively before other retinal capillary networks [3–5].

Advances in retinal imaging, namely adaptive optics scanning light ophthalmoscopy [6–8] and optical coherence tomography angiography (OCT-A) [9–15], have allowed *in vivo* visualization of RPCs in humans at a level previously achievable only with histology. Recent cross-sectional studies using OCT-A have shown that RPC density is indeed decreased in glaucomatous [16–22] and nonglaucomatous optic neuropathies [23–26], as well as in branch and central retinal vein occlusions [27, 28]. Of note, the majority of these studies have defined RPC density as the perfused vascular density within the OCT-A RPC layer, between the inner limiting membrane and the posterior boundary of the retinal nerve fiber layer (RNFL). This layer includes not only the superficial capillary plexus but also deeper capillaries and large peripapillary vessels. [29]

Two possibilities for the pathophysiology of the observed changes to the RPC layer have emerged. In the first, decreased blood flow detectable as decreased RPC density occurs first, causing ischemia of nerve fibers and RNFL thinning [30–32]. In the second, RPC loss occurs via a neurovascular coupling mechanism, secondary to loss of surrounding RNFL from a primarily neuropathic process [26]. Both mechanisms may be in play to different degrees in the various retinal and optic nerve pathologies. It is thus important to continue to develop RPC metrics as they may potentially be sensitive and specific biomarkers in the diagnosis and prognosis of disease.

Studying age-related changes to the RPCs is important since it may facilitate the differentiation of pathologic processes from age-related changes [33, 34]. OCT-A has recently revealed a decrease in macular microvascular density with age in otherwise healthy individuals [35–38]. Previous studies have shown that RNFL thickness decreases with age in otherwise healthy individuals [39–43]. Given the close interdependence between RNFL thickness and RPC density, we would expect RPC density to decrease with age as well. There is limited data on the effect of aging on RPC density, however. To the best of our knowledge, only one study has looked at RPC density in relation to age in healthy eyes, and has found no correlation [44].

Studying age- and disease-related changes to the large peripapillary vessels (noncapillary vessels; LPVs) is important as well. Previous studies have demonstrated that arteriolar diameters narrow during aging and hypertension [45–47]. Since OCT-A vascular images represent the perfused intraluminal space, LPV density may decrease with age. It is important to study the changes in RPC and LPV density separately, since changes may occur along different trajectories, and one may prove more vulnerable than the other.

The purpose of this study was to expand upon the healthy control reference database of RPC density, and to present an early reference database for LPV density. We also set out to explore the changes in RPC density and LPV density that occur with aging. Additionally we introduce a deviation mapping technique, based on age-matched RPC density group means and standard deviations (SDs), to qualitatively and quantitatively assess the significance of regional RPC density changes in different diseases.

## Methods

### Study population

This was a multicenter study, conducted at the New York Eye and Ear Infirmary of Mount Sinai and at the Medical College of Wisconsin. This study followed the tenets of the Declaration of Helsinki and was approved by the Institutional Review Boards of both New York Eye and Ear Infirmary of Mount Sinai and the Medical College of Wisconsin. Written informed consent was obtained from the subjects after explanation of the nature and possible consequences of the study.

We examined 133 healthy control eyes (133 subjects, 74 female, 59 male; 76 right and 57 left eyes; mean age 41.5 yrs, range 11–82 yrs; Table 1). Four eyes with different pathologies–primary open angle glaucoma (POAG), proliferative diabetic retinopathy (PDR), retinal vein occlusion (RVO) and sickle cell retinopathy (SCR)–were chosen.

**Table 1:** Number of eyes and % male eyes per each decade of life included in this study.

	Overall	10-19yrs	20-29yrs	30-39yrs	40-49yrs	50-59yrs	≥60yrs
N of eyes	133	12	31	23	17	30	20
Sex (Male %)	44%	17%	52%	57%	41%	47%	35%

### Subject selection

The healthy controls had clear media and no known retinal or optic nerve head pathology, confirmed by fundus photography and OCT optic nerve head and macula scans (Zeiss Visucam, Carl Zeiss Meditec Inc, Dublin, California; Zeiss Cirrus HD-OCT, Carl Zeiss Meditec Inc, Dublin, California; Heidelberg Spectralis HRA+OCT, Heidelberg Engineering Inc, Heidelberg, Germany). A self-reported negative past medical history, including no cardiovascular disease such as hypertension or diabetes mellitus, was also required. If images were available for both eyes of a given control, the decision as to which eye to include in the study was made based on image quality; or, randomly, in the cases where images of both eyes were comparable in image quality. The diseased eyes included were only required to have good central fixation and clear media.

### Image acquisition

A commercial spectral-domain OCT-A system (Avanti RTVue-XR, Optovue, Fremont, California) was used to obtain nominal 4.5x4.5mm *en face* peripapillary scans using a wavelength of 840nm and axial line rate of 70kHz. The system’s split-spectrum amplitude decorrelation angiography (SSADA) algorithm was used to map the perfused vessels for each scan [48].

### Image analysis

#### Image processing

**Fig 1:**
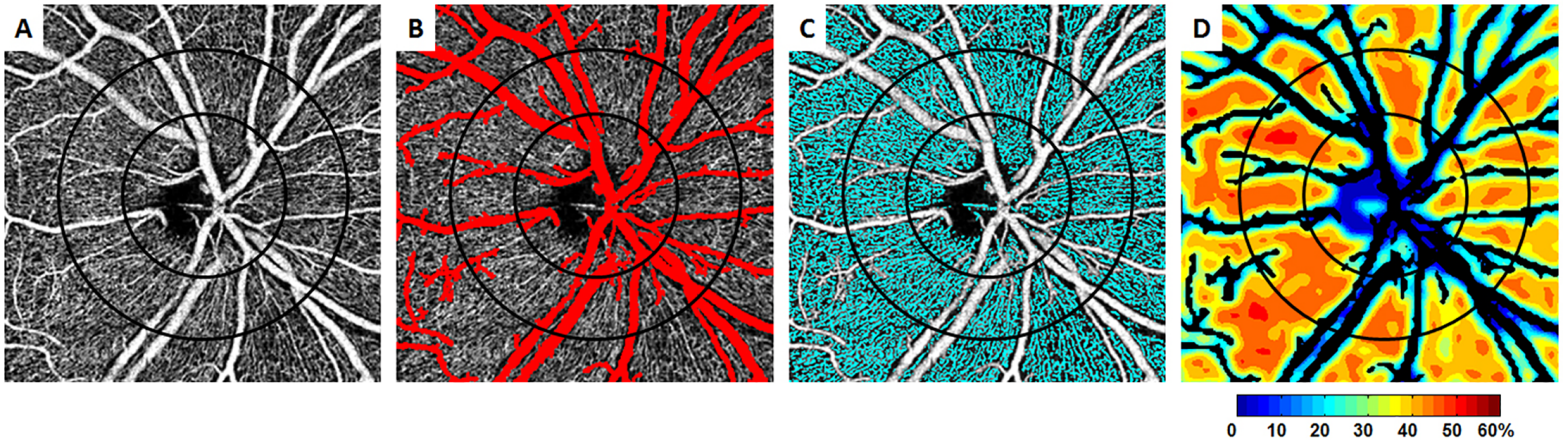
Creating an RPC density map for an individual eye. A) A contrast-stretched 4.5x4.5mm *en face* peripapillary OCT-A scan at the RPC layer was used. An annular ROI measuring 0.75mm in width was created with two concentric circles centered at the optic nerve head. B) Large peripapillary vessels were extracted, outlined in red. C) Capillaries were segmented, outlined in cyan. D) The resulting RPC density map. Areas involving large peripapillary vessels appear in black.

Image processing targeted the OCT-A RPC layer, which was defined as the layer between the inner limiting membrane and the posterior boundary of the RNFL. Our initial image processing procedures were previously described by Scripsema et al [19]. In brief, a peripapillary OCT-A scan of the RPC layer (Fig 1A) was used for global thresholding to create a binary image, enabling the extraction of the LPVs (Fig 1B). Next, local thresholding was used for capillary segmentation (Fig 1C). A color-coded RPC density map was created after the removal of the LPVs (Fig 1D).

Our methodology represents nominal values and no axial length correction was performed. Using the Optovue system software (AngioVue version 2015.100.0.35, Optovue Inc, Fremont, California), the center of the optic disc was marked based on the automatic optic disc margin determination [49]. The center of the optic disc was used to generate two concentric circles with diameters of 1.95- and 3.45-mm, creating a 0.75mm-wide annular region of interest (ROI) used for quantitative RPC and LPV density analysis (Fig 1). RPC density (%) was calculated as the RPC pixel area divided by the difference between total annular ROI pixel area and LPV pixel area. LPV density (%) was calculated as LPV pixel area divided by the total annular ROI pixel area. For further analysis, the annular ROI was subdivided into four quadrants–temporal, nasal, superior and inferior.

$$RPC\;density,\%=\frac{RPC\;pixel\;area}{ROI\;pixel\;area-Large\;peripapillary\;vessel\;pixel\;area}\times 100\%$$

$$LPV\;density,\%=\frac{Large\;peripapillary\;vessel\;pixel\;area}{ROI\;pixel\;area}\times 100\%$$

#### Statistical analysis

Linear regression analysis was performed on all 133 controls, and one-way ANOVA and pairwise Tukey-Kramer tests were performed between each decade of life (10-19yr, 20-29yr, 30-39yr, 40-49yr, 50-59yr, 60yr or older) using annular and quadrant RPC and LPV density values to evaluate for the impact of aging. A two-tailed unpaired t-test was performed to evaluate for the impact of sex on RPC and LPV density. P values less than 0.05 were considered statistically significant.

#### Group mean and SD RPC density maps

Group mean and SD RPC density maps were created for each decade of life. First, all left eye OCT-A RPC density maps were flipped horizontally so that the temporal aspect of the optic disc was located on the left in both left and right eye images. RPC density maps in a given age group were then aligned and averaged based on the estimated optic disc center, creating a group mean and SD RPC density map for each decade of life. Regions of extracted LPVs were excluded from computation in these density maps.

#### Deviation mapping

**Fig 2:**
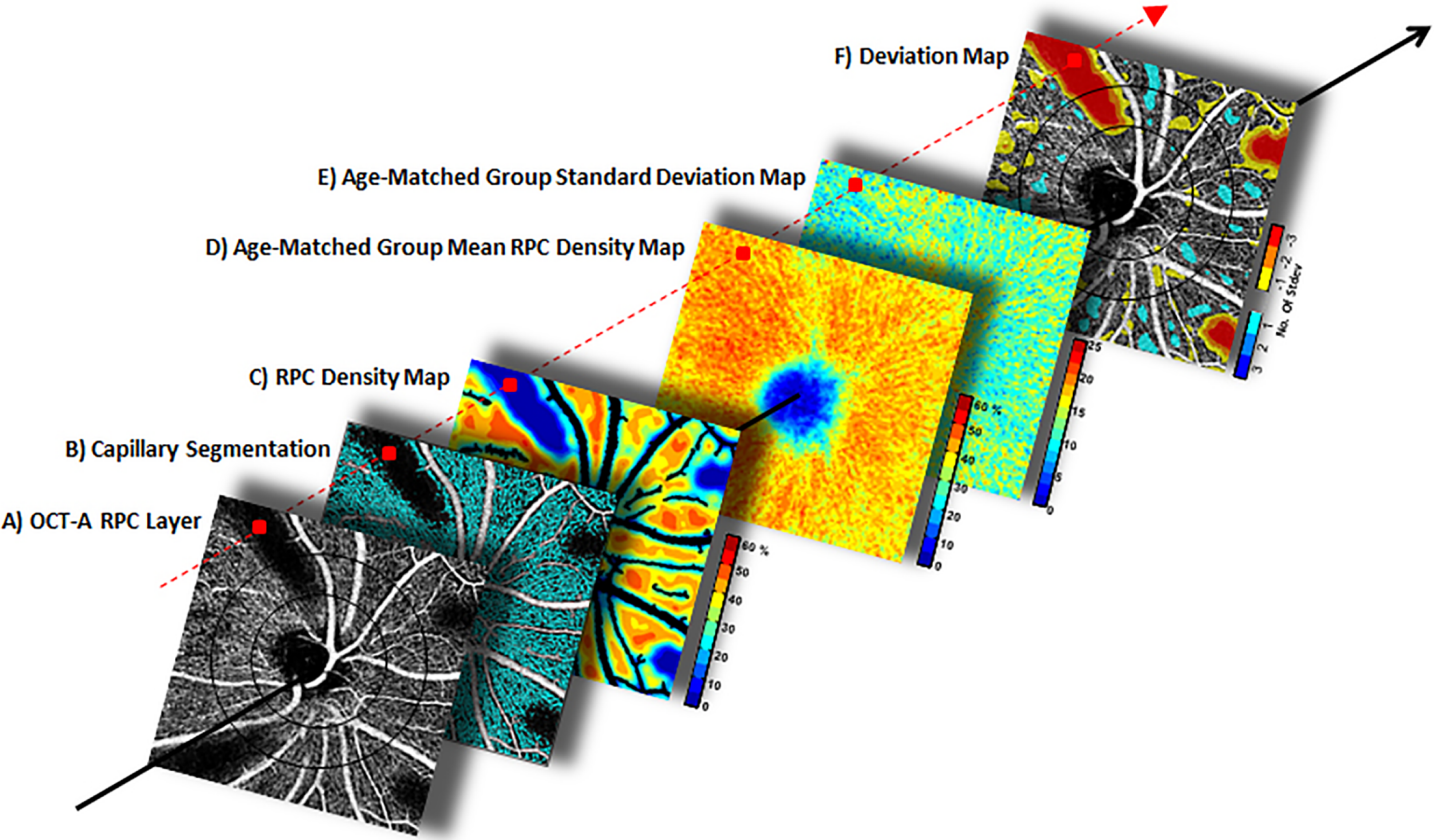
Deviation mapping methodology explained with simulated capillary defects. A-C) Creating an RPC density map for the tested eye. D & E) The individual RPC density map was then compared against the age-matched group mean RPC density and SD maps. F) In the deviation maps, cooler colors indicate regions with above group mean densities, and warmer colors below group mean densities. On the scale, darker shades of blue indicate higher densities (cyan indicates 0.5–1.4, light blue indicates 1.5–2.4, and dark blue indicates 2.5 and above). On the other hand, darker shades of red indicate lower densities (yellow indicates 0.5–1.4, orange indicates 1.5–2.4, and red indicates 2.5 and above).

Using age group mean and SD RPC density maps, deviation maps for a given individual eye were created. For the purposes of demonstrating our methodology, we used a 4.5x4.5mm *en face* optic disc OCT-A scan at the RPC layer that has had areas of the capillary bed artificially erased (Fig 2A). Fig 2A–2C illustrate the creation of an individual RPC density map. This individual RPC density map was then compared against an age-matched group mean RPC density map (Fig 2D) and corresponding SD map (Fig 2E). At a given locus (Fig 2, red squares), the normative group mean (Fig 2D) was subtracted from the individual RPC density value (Fig 2C), and the resulting value was divided by the respective group SD value (Fig 2E) to produce a color coded deviation map (Fig 2F). The region within the 1.95 mm circle was excluded from deviation mapping due to high inter-subject RPC density variability within this region.

## Results

The overall and group mean±SD of annulus and quadrant RPC and LPV densities are shown in Table 2.

**Table 2:** Overall and age group mean±SD of annulus and quadrant RPC and LPV densities.

	Annulus	Temporal	Nasal	Superior	Inferior
**Table 2A. RPC density (%)**					
Overall	42.5±1.47	43.0±2.09	42.6±2.26	41.8±1.81	42.6±1.82
**10-19yrs**	43.1±0.669	44.2±1.12	42.7±0.954	42.2±1.24	43.2±1.46
**20-29yrs**	42.7±1.10	43.6±1.71	42.7±1.63	42.0±1.49	42.5±1.47
**30-39yrs**	42.7±1.11	43.0±2.06	42.7±2.07	41.8±1.44	43.2±0.944
**40-49yrs**	43.1±1.05	43.3±1.77	43.4±1.38	42.3±1.70	43.2±1.27
**50-59yrs**	42.7±1.31	42.9±1.81	43.0±2.02	42.3±1.84	42.6±2.19
**≥60yrs**	40.8±2.05	41.0±2.59	40.9±3.76	40.3±2.25	41.0±2.19
**Table 2B. LPV density (%)**					
Overall	21.5±3.07	16.7±5.42	18.1±5.28	25.4±4.69	25.7±4.67
**10-19yrs**	22.4±2.80	15.4±3.89	20.3±3.20	26.5±5.25	27.3±3.65
**20-29yrs**	21.9±2.70	15.8±4.72	18.1±5.54	26.5±3.26	27.1±4.08
**30-39yrs**	21.5±3.02	16.7±6.26	18.5±4.71	25.7±4.76	25.2±3.32
**40-49yrs**	22.6±2.91	18.3±5.98	19.1±4.59	26.3±4.06	26.5±3.41
**50-59yrs**	21.2±2.85	17.6±6.00	17.9±5.14	24.3±5.46	25.1±5.03
**≥60yrs**	19.7±3.73	16.0±4.83	15.6±6.67	23.4±4.98	23.7±5.74

### RPC density

**Fig 3:**
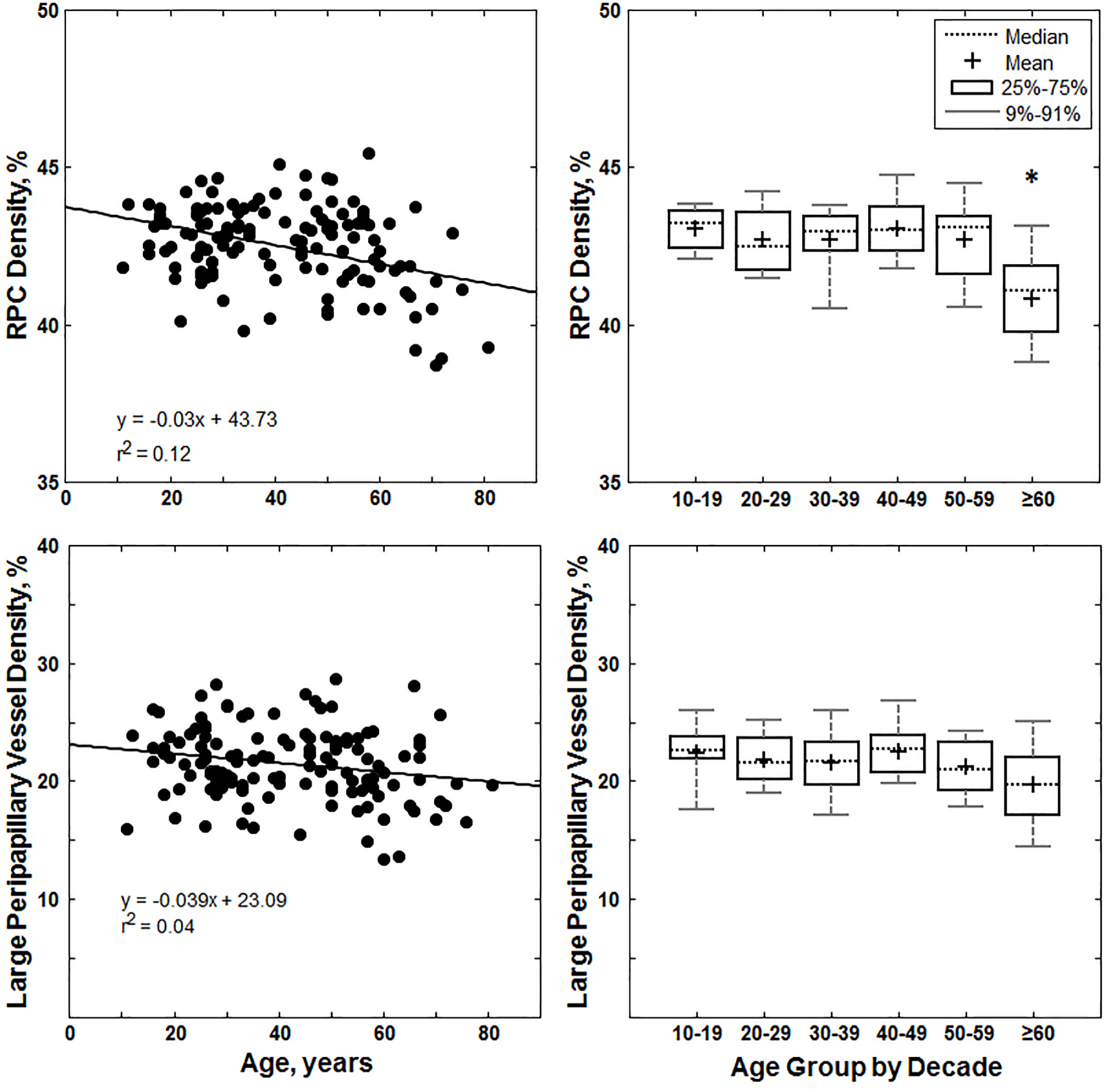
Top row: Linear regression and box plot for annular RPC density versus age for the 133 healthy controls. In the box plot, the asterisk indicates a significant difference in RPC density in the ≥60yr age group compared to that of all the younger age groups. Bottom row: Linear regression and box plot for annular large peripapillary vessel density versus age for the 133 healthy controls.

Average RPC density across all healthy control subjects was 42.5±1.47%. Our linear regression models indicated that annular RPC density decreases with age (p = 0.0149, see Fig 3 top row for regression slope). However, in comparing RPC density across the age groups using ANOVA and pairwise Tukey-Kramer tests, only the ≥60yr group showed a significantly lower RPC density compared to that in each younger decade of life (p<0.01 for all comparisons). RPC density did not differ significantly with sex in either annulus or quadrant analysis (annulus analysis: female 42.6±1.5% vs male 42.3±1.5%; p = 0.27).

**Fig 4:**
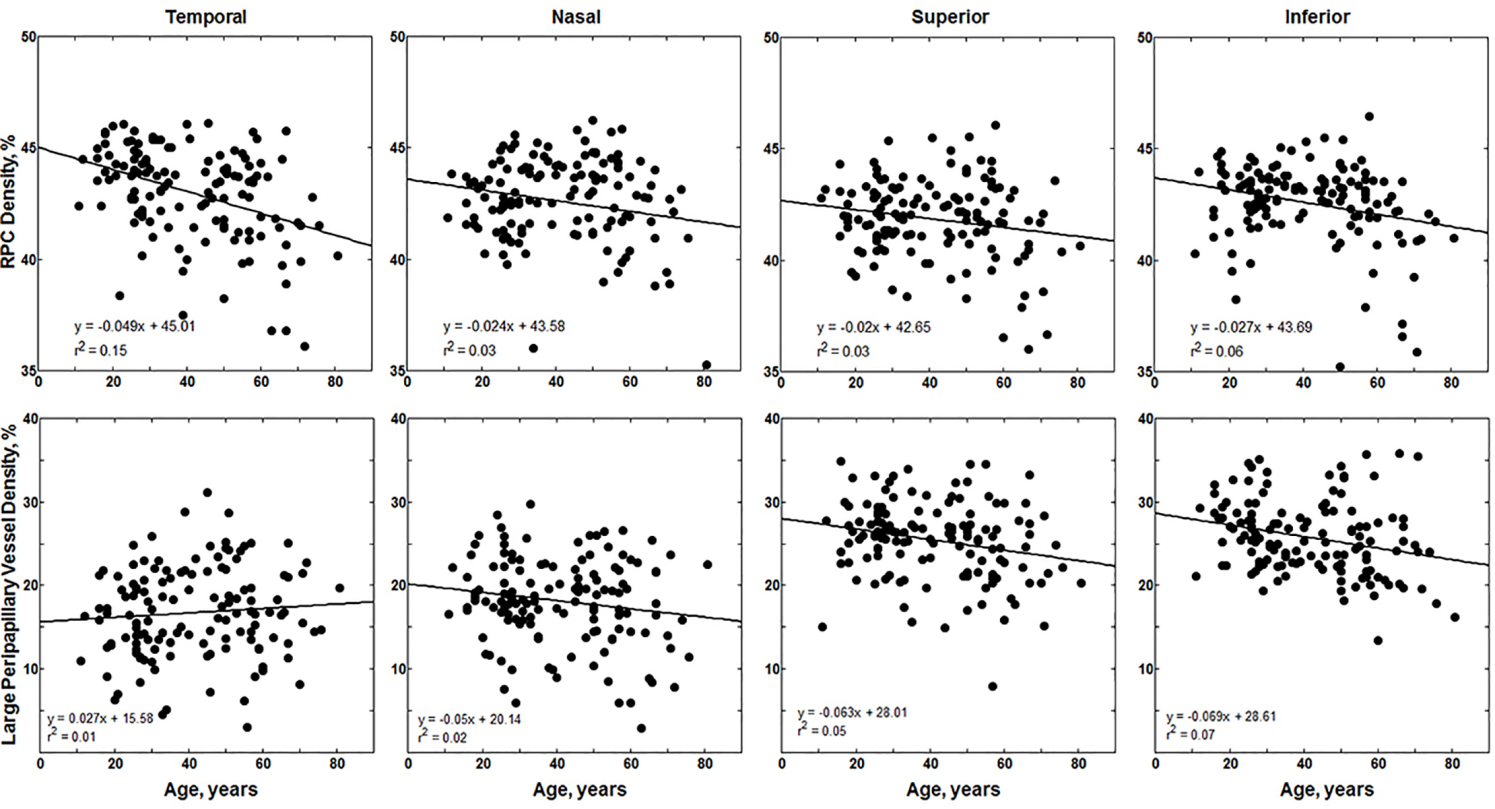
Top row: Linear regression for RPC density per quadrant versus age for the 133 healthy controls. Bottom row: Linear regression for LPV density per quadrant versus age for the 133 healthy controls.

A similar relationship between RPC density and age was observed in the quadrant analysis of RPC density (Fig 4 top row). The temporal quadrant decreased the most significantly with age (p<0.0001), followed by the inferior (p = 0.0035), the superior (p = 0.0342), and the nasal quadrant (p = 0.0406; see Fig 4 top row for regression slopes). Furthermore, the RPC density at the temporal quadrant was the highest with an average of 43.0±2.09%, followed by the inferior quadrant (42.6±1.82%), nasal quadrant (42.6±2.26%), and superior quadrant (41.8±1.81%).

### LPV density

Average LPV density across all healthy control subjects was 21.5±3.07%. Our linear regression models indicated that annular LPV density decreased significantly with age (p = 0.0149, see Fig 3 bottom row for regression slopes). Quadrant linear regression analysis showed that LPV density decreased with age most significantly in the inferior (p = 0.0027) and superior quadrants (p = 0.0149, see Fig 4 bottom row for regression slopes). ANOVA and pairwise Tukey-Kramer tests showed a trend towards decreasing density after age 60; however, these differences were not significant in either annulus or quadrant analysis (p>0.05). Furthermore, LPV density did not show a significant difference between sex in either annulus or quadrant analysis (annulus analysis: female 21.1±3.22% vs male 21.9±2.82%; p = 0.14).

### Group mean and SD RPC density maps

**Fig 5:**
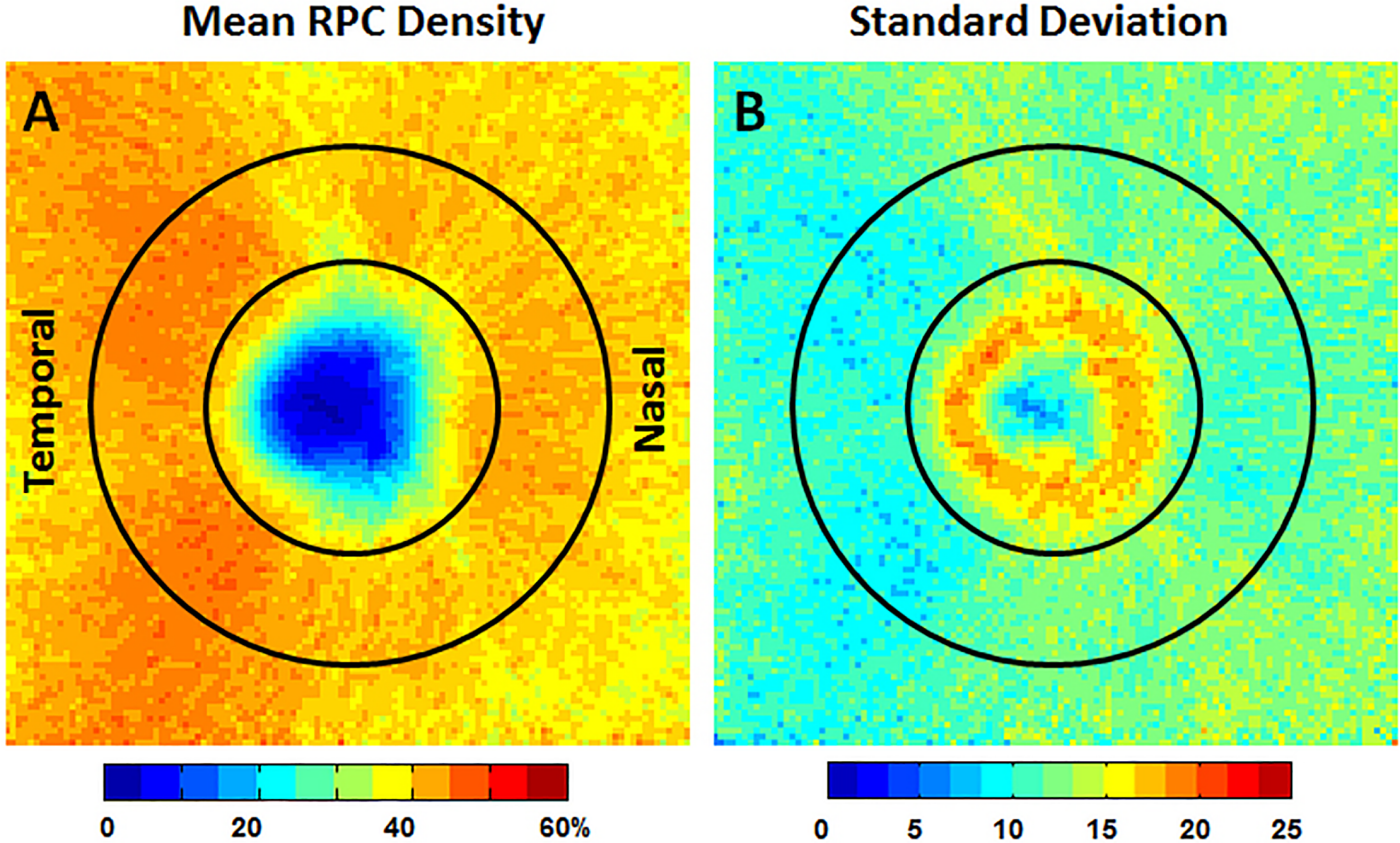
A) Mean RPC density and B) SD maps for all 133 healthy controls. The relatively lower mean RPC density associated with slightly higher SDs in the superior and inferior regions was due to the presence of periarteriolar capillary-free areas after the removal of large peripapillary vessels. A relatively low RPC density and high SD was observed within the 1.95 mm optic disc margin, suggesting high inter-subject variability of RPC density within the optic disc (Fig 5B). For this reason, RPC density data within the optic disc margin was not used for deviation mapping. The temporal aspect of the optic disc is to the left in all images.

**Fig 6:**
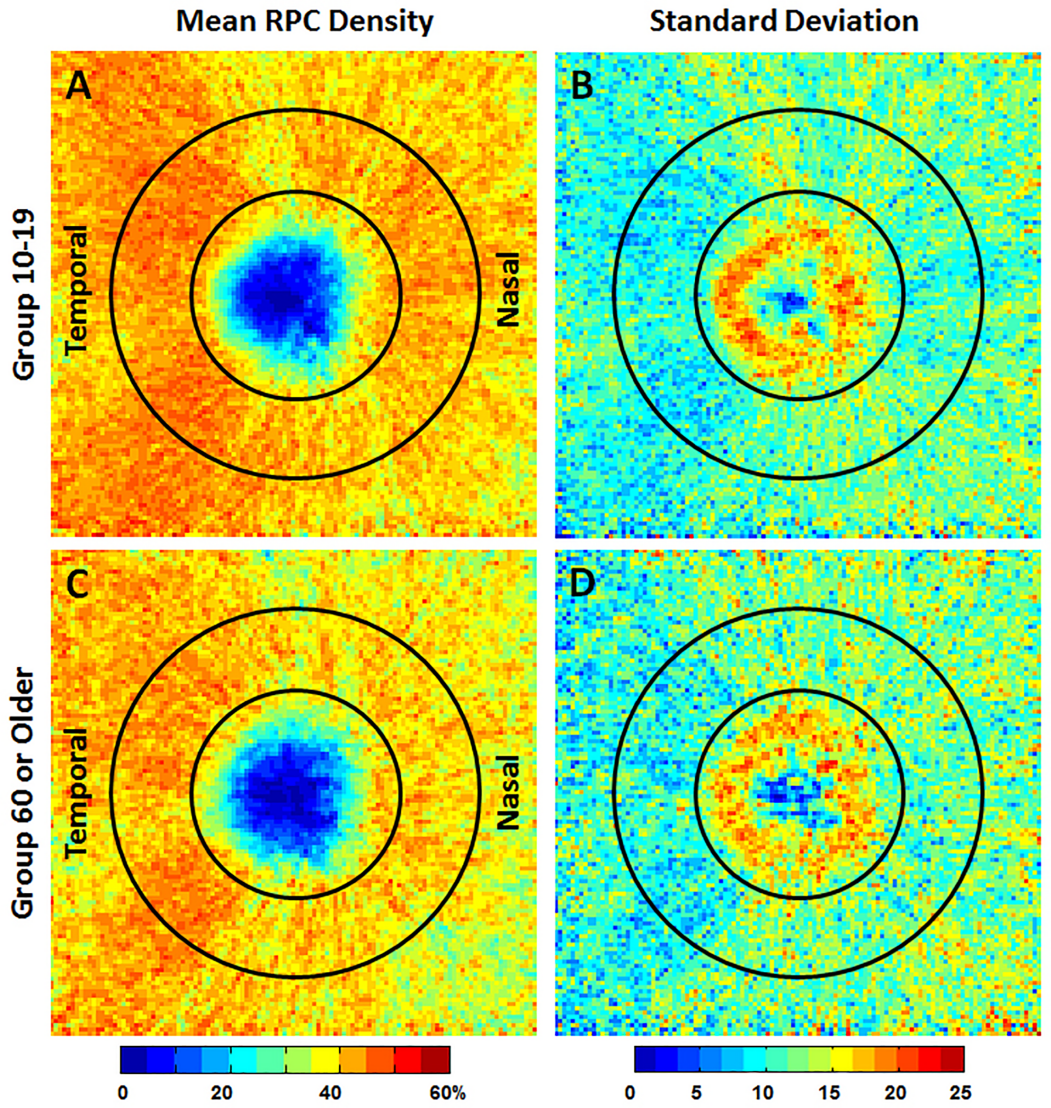
A & C) Group mean RPC density and B & D) SD maps for two different age groups, the 10-19yrs and the ≥60yrs age group. Relatively lower RPC density was observed in the ≥60yrs age group. The temporal aspect of the optic disc is to the left in all images.

The group mean RPC density map of the 133 healthy controls shows a relatively dense RPC along the superotemporal and inferotemporal regions, consistent with known anatomy of denser nerve fibers in these regions (Fig 5). A comparison of group mean and SD RPC density maps between the youngest (10-19yrs) and oldest (≥60yrs) age groups is shown in Fig 6. While there was a general attenuation of the RPC density in the ≥60yrs age group, the group SD was similar between groups.

### Deviation mapping in disease eyes

**Fig 7:**
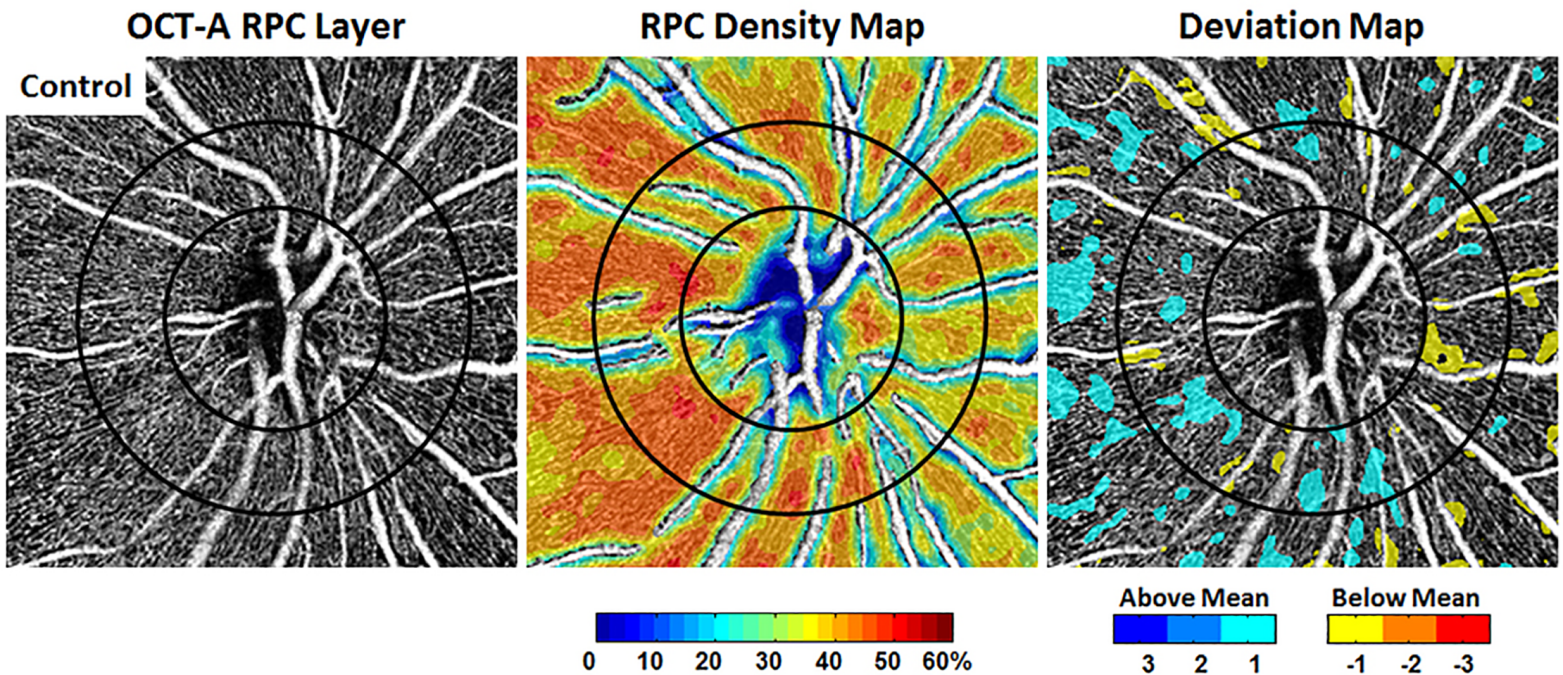
RPC density mapping versus deviation mapping on a healthy control eye. In the deviation map, cooler colors signify number of SDs above the age-matched group mean density for a given locus, and hotter colors signify below. The temporal aspect of the optic disc is to the left in all images.

**Fig 8:**
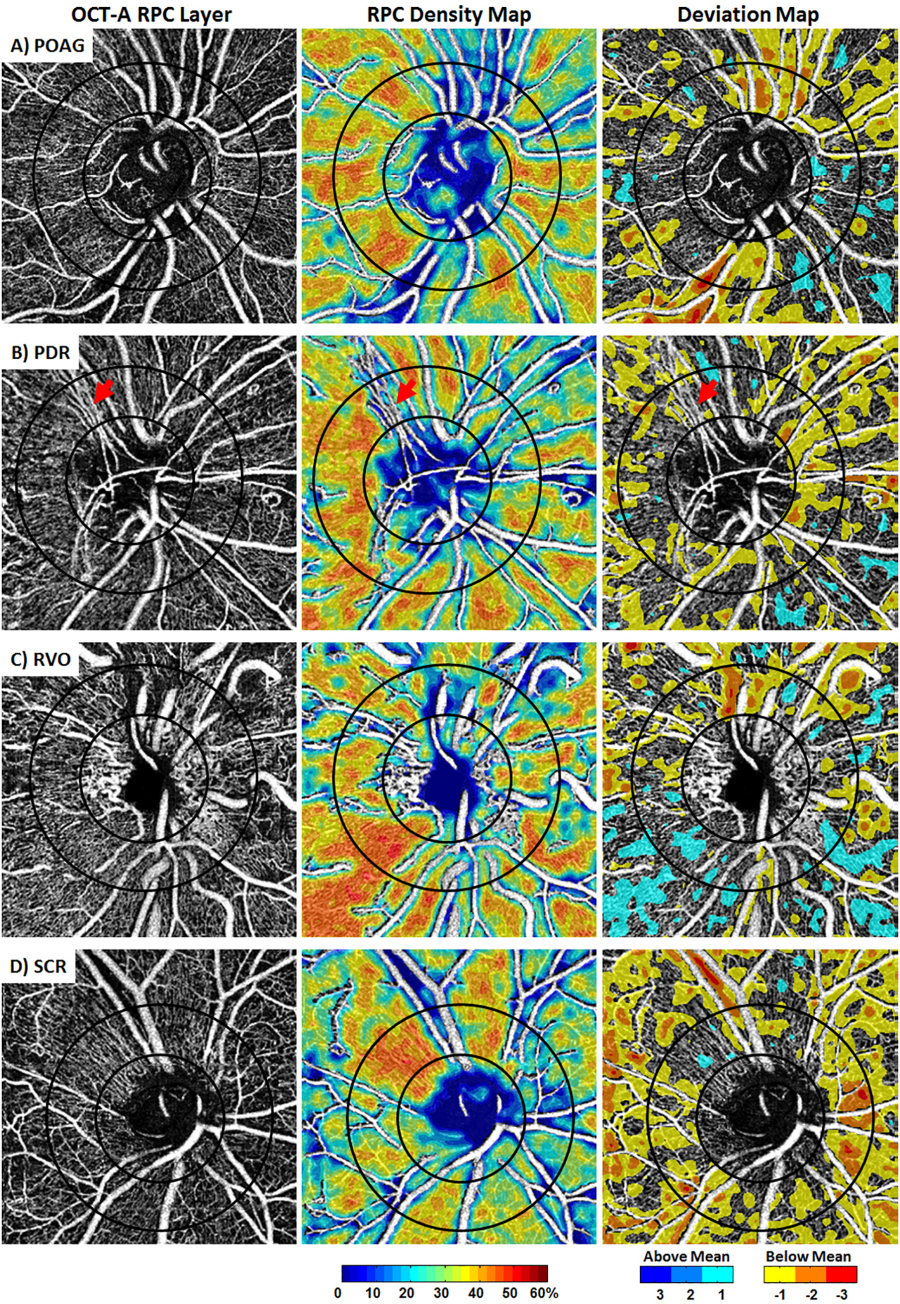
**Individual RPC density mapping vs deviation mapping in A) POAG, B) PDR, C) RVO, D) SCR.** Deviation maps highlight the areas with RPC density below or above the age-matched group mean, allowing for the visualization and identification of focal areas of significant change. In the PDR eye, the neovascular vessels emanating from the superior-temporal aspect of the disc have been interpreted by our software as large vessels due to the dilated vessel diameter, and have been removed from the analysis (red arrows). The temporal aspect of the optic disc is to the left in all images.

Figs 7 and 8 are examples of the application of deviation mapping to a healthy control eye and 4 different disease eyes, respectively. The RPC density maps enable visualization of regional variations in density, but do not give information on the significance of this variation. The deviation maps are able to show how significant the variation is relative to the respective age-matched norm.

## Discussion

Here we showed that RPC density shows significant attenuation after age 60 years in otherwise healthy individuals. Our analysis showed that the temporal quadrant had not only the highest RPC density compared to the other quadrants and annulus, but also showed the greatest amount of change with age. The temporal and nasal quadrants showed greater significant RPC density change with age compared to the annular RPC density, implying that these quadrants may be more sensitive compared to annular analysis in detecting changes with age. Previously published studies on age-related thinning of the RNFL differ from one another on which quadrant shows the most changes with age, but none of them report that it is the temporal quadrant that shows the most change [39–41]. It is important to remember that most of these studies included LPVs in their RNFL thickness measurements, limiting comparison to our results.

Our linear regression analysis indicated that LPV density decreases with age in otherwise healthy individuals, being most significant in the inferior and superior quadrants. Our analysis of variance results must be interpreted with caution since the trend of LPV density decreasing with age did not reach statistical significance. Anatomically we know that the inferior and superior quadrants are the location of the main large vessels after the bifurcation of the central retinal artery and vein within the optic nerve head. Previous studies have reported an age-dependent reduction in major retinal artery and vein caliber [45, 46, 50], and an age-dependent increase in retinal vessel wall thickness, particularly arterial wall thickness [51]. These studies are in agreement with our results, since a smaller caliber and increased vessel wall thickness translates to decreased intraluminal perfused area. Our results indicate that LPV density may be an important biomarker in studying disease progression and response to treatment.

Our results underscore the importance of developing a more robust age-matched reference RPC and LPV density database, in order to perform more sensitive and precise analysis of diseased eyes. One other manuscript has looked at RPC density changes as they relate to aging [44]. The study by Mansoori et al looked at 52 healthy individuals aged 19 to 63 years old and found no changes in RPC density with aging. These results largely corroborate with ours since in our study, significant RPC density changes were observed only after the age of 60. The study had a notable difference from our study. Their definition of the RPC layer differed slightly from ours. They defined the RPC layer as from the inner limiting membrane to 100 microns below the inner limiting membrane, while we defined it as to the posterior boundary of the RNFL.

In this study, we also illustrate the benefit of deviation mapping based on age-matched reference data in assessing for the significance of regional RPC variation in disease states. It is important to continue to develop methodology of RPC analysis, as a few early studies have already demonstrated the potential for RPC density as a useful biomarker for detecting and monitoring disease. Using OCT-A, Holló et al demonstrated *in vivo* the dynamic relationship between increased IOP and decreased perfused peripapillary capillary density in treatment-naïve patients with high IOP, and showed that large medical IOP reduction may result in a clinically significant increase in peripapillary capillary perfusion [52]. Chen et al recently showed that in eyes with glaucoma and single-hemifield visual field loss, the peripapillary microvascular density of the normal hemisphere was greater than that of the abnormal hemisphere, but was less than that of a normal control eye [32]. RNFL thickness comparisons proved less sensitive, showing similar RNFL thicknesses between the normal hemispheres of glaucomatous eyes and normal control eyes.

Our study had a number of limitations. Although our results showed a significant RPC density decrease after the age of 60 years, our sample size was limited. It is important for future studies to recruit more healthy control eyes and expand on the age-, sex- and race-matched reference RPC density database. The box-plot for RPC density appears to show a sudden decrease in density after the age of 60 years, but this may be an effect of our grouping method. A larger n after the age of 60 years would allow the data to be divided into decades for a closer analysis of RPC changes after the age of 60 years. In this study, all past medical histories were self-reported and we did not collect vital signs or check any blood chemistry. Thus, some patients may have had undiagnosed or unreported cardiovascular disease including diabetes mellitus and hypertension. Furthermore, with increasing age, media opacities in the cornea, lens and/or vitreous become more prevalent. Although they may not have appeared significant, they may have influenced image quality in the older controls, leading to underestimation of RPC density values.

Our image analysis methodology has some limitations. As mentioned before, we define RPC density as perfused capillary density within the Optovue-defined RPC layer between the inner limiting membrane and the posterior boundary of the RNFL. The catchment area at these depths may involve some of the deeper capillary plexuses, causing an overestimation of RPC density measurements. However, we believe that as long as the same methodology of image acquisition is used to acquire data across the board, our results and conclusions remain valid.

In this study we did not perform axial length correction since vessel density was computed as an area-to-area ratio (RPC pixel area divided by ROI pixel area). Still, without axial length correction, the sampled area that density is integrated over is slightly different in each eye. Furthermore, it has been reported that retinal microvascular density is decreased in myopes compared to emmetropes [53]. Future studies should categorize eyes with myopia as a subset of the normal population. Another limitation in our methodology was in analyzing regions with neovascularization. Peripapillary neovascularization, if significant enough, is misinterpreted by our software as large blood vessels, and is taken out of the analysis as shown by Fig 8B (red arrows). Thus, instead of showing increased density in an area of neovascularization, our density maps show no density data.

Future studies should explore the use of deviation mapping based on normative reference data clinically, and correlate it to current clinical tools, both cross-sectionally for earlier diagnosis of disease, and longitudinally for monitoring disease and/or assessing adequacy of therapy.

## Supporting information

S1 File Excel datasheet. Please refer to this document for LPV and RPC density values per annulus and quadrant for each individual participant in this study.
(XLSX)
